# Cell cycle-independent roles of p19^INK4d^ in human terminal erythropoiesis

**DOI:** 10.1186/s40880-017-0189-4

**Published:** 2017-02-23

**Authors:** Xu Han, Jing Liu

**Affiliations:** The State Key Laboratory of Medical Genetics & School of Life Sciences, Central South University, Changsha, 410078 Hunan P. R. China

Normally, cyclin interacts with cyclin-dependent kinase (CDK) to form a cyclin-CDK complex, which promotes cell cycle progression, whereas cyclin-dependent kinase inhibitor (CDKI) molecules inhibit the formation of cyclin-CDK complex, arresting cell cycle. Terminal erythropoiesis is closely coordinated with cell cycle exit, which is regulated by cyclins, CDKs, and CDKIs [1]. In the global transcriptome of human terminal erythropoiesis [2], p19^INK4d^ is expressed highly, and its expression is significantly up-regulated during human terminal erythropoiesis. However, the roles of p19^INK4d^ in terminal erythropoiesis are still unknown.

As reported in our article recently published in *Blood* entitled “Unexpected roles for p19^INK4d^ in posttranscriptional regulation of GATA1 and modulation of human terminal erythropoiesis” [3], we demonstrated what roles p19^INK4d^ plays in human terminal erythropoiesis. We found that, in the erythropoietin-induced, CD34-positive hematopoietic stem cell (HSC) differentiation system, knockdown of p19^INK4d^ delays terminal erythroid differentiation, inhibits erythroblast growth due to increased apoptosis, and leads to the generation of abnormally nucleated late-stage erythroblasts. Unexpectedly, knockdown of p19^INK4d^ did not affect cell cycle, and these functions caused by p19^INK4d^ knockdown were via decreasing levels of GATA-binding protein 1 (GATA1). Furthermore, we found that p19^INK4d^ modulates GATA1 protein levels through a novel pathway, the phosphatidylethanolamine-binding protein 1 (PEBP1)-phosphorylated extracellular signal-regulated kinase (*p*ERK)-heat shock 70 kDa protein (HSP70)-GATA1 pathway [3].

As a classical CDKI member, p19^INK4d^ has been shown to inhibit the formation of cyclin D-CDK4/6 complex, arresting cell cycle in the G_0_/G_1_ phase [4]. p19^INK4d^ was often induced to inhibit the proliferation of many kinds of tumor cells, such as T cell acute lymphoblast leukemia cells and lung cancer H1299 cells [4, 5]. Additionally, deletion of p19^INK4d^ leads to the development of many tumors, including osteosarcomas [6] and anterior lobe tumors [7]. p19^INK4d^ is also involved in HSC quiescence, megakaryocyte differentiation, and granulocytic differentiation, which are closely associated with cell cycle arrest [8–10]. However, as shown in our study, p19^INK4d^ played important roles independent of cell cycle regulation, and the lack of cell cycle change was probably due to the compensatory up-regulation of p18^INK4c^ following p19^INK4d^ knockdown. Our findings provide greater understanding of the role that CDKIs play in cell cycle regulation.

In conclusion, our study revealed the cell cycle-independent roles of p19^INK4d^ in human terminal erythropoiesis via a novel PEBP1-*p*ERK-HSP70-GATA1 pathway. These findings will likely improve understanding of disordered erythropoiesis, including thalassemia, myelodysplastic syndrome, and congenital dyserythropoietic anemia, and guide future studies that focus on CDKIs.
